# Global scale high-resolution habitat suitability modeling of avifauna providing pollination service (sunbirds, Nectariniidae)

**DOI:** 10.1038/s41598-025-85587-x

**Published:** 2025-03-19

**Authors:** Masoud Yousefi, Michaël P. J. Nicolaï, Luciano Bosso, Anooshe Kafash, Bagher Nezami, Eskandar Rastegar-Pouyani

**Affiliations:** 1 Faculty of Governance, University of Tehran, Tehran, Iran; 2 Biology Department, Evolution and Optics of Nanostructures Group, Ghent University, Ghent, Belgium; 3 Institute for Agriculture and Forestry Systems in the Mediterranean, National Research Council of Italy, Piazzale E. Fermi, 1, Portici, 80055 NA Italy; 4 Department of Biology, Hakim Sabzevari University, Sabzevar, Iran; 5 Research Group of Biodiversity & Biosafety, Research Center for Environment and Sustainable Development, Tehran, Iran

**Keywords:** Avian distribution, Conservation, Ecosystem service, Maxent, Pollination, Species distribution models, Ecology, Biodiversity, Macroecology

## Abstract

Avian species provide important ecosystem services such as nutrient cycling, seed dispersal, meat provision, pest control, scavenging, and pollination. Currently, the populations of avian pollinators are declining due to climate change and human impact, and it is crucial to identify species-rich areas for their conservation. Sunbirds (Nectariniidae) are important vertebrate pollinators with a wide distribution that include Africa, Asia and Australasia. Here, we assembled distribution records of sunbird species and applied a maximum entropy approach to model sunbird habitat suitability in the world. We also quantified sunbirds composition similarity among the terrestrial biomes. We found that sunbird habitat suitability reached a peak in Southeast Asia, and in western and central parts of the African continent. Sunbird richness was highest in the Tropical and Subtropical Moist Broadleaf Forests biome. Solar Radiation Index (SRI), precipitation of the warmest quarter, and human footprint index were the most important predictors of sunbirds global habitat suitability. Geographic regions identified to have the highest suitability and richness for sunbirds have high priority for conservation of this unique group of avian pollinators and the ecological services they provide.

## Introduction

Human well-being depends on ecosystem services provided by biodiversity^1–4^. Water and air quality regulation, medicinal resources, biological control, recreation and mental and physical health, erosion prevention, and maintenance of soil fertility and pollination are some examples of ecosystem services provided by nature^1,4^.

Pollinators play a critical role in nature and provide ecological services to humans^1–7^. For instance, pollinator species affected about 87.5% of the world’s flowering plants and 75% of the world’s major crop^6,8^. Plant species pollinated by animals are frequently used for medicines, food, and construction materials^9^. Ratto et al.^5^ showed that in the absence of vertebrate pollinators, fruit and seed production can be reduced by 63%, highlighting the importance of vertebrate pollination. Despite their interest to human well-being, pollinator abundance and diversity are declining around the globe due to habitat loss, land degradation and fragmentation, climate changes, invasive alien species, hunting, and fire^5,10–13^. In this context, identifying where pollinator habitat suitability is highest can help their conservation planning.

Avian species provide important ecosystem services such as nutrient cycling, seed dispersal, meat provision, pest control, scavenging, and pollination^5,14,15^. Birds are important pollinators and are essential for the life cycle of a significant proportion of cultivated and wild plant species^5,14,15^, including those belonging to the families Nectariniidae, Trochilidae, Meliphagidae, and Loridae^5,16^. Sunbirds (Nectariniidae) are a group of avian species that are distributed over Africa, Southern Asia, and parts of the Australasia region^16^. They are important pollinators across their global distribution range^7,17^. For example, it is well known that in Africa, sunbirds are the dominant vertebrate pollinator^17^. Newmark et al.^7^ showed that across Africa, 68% of the 329 genera and 44% of the 468 species of sunbirds’ known food plants are used by humans for medicine, food, building materials, or other uses. Despite sunbirds’ significant role in pollination across their distribution range, their global habitat suitability and richness remain relatively uninvestigated^7,17^. Thus, it is important to identify areas in which pollinator richness reaches high value of habitat suitability.

Species Distribution Models (SDMs) are frequently used in different research areas such as paleoecology, evolution, health geography and in particular in biogeography and conservation^18–22^. These models use species occurrence data and environmental variables such as climate, topography and vegetation, for estimating the habitat suitability in time and space^18,23^. SDMs have been used to identify hotspots biodiversity and priority areas for conservation^24–27^, habitat suitability^28^ and to assess the impacts of climate change on biodiversity^28–31^. For instance, de Carvalho et al.^25^ modeled the habitat suitability of 24 threatened and endemic bird species using SDMs in Brazil. Then they stacked the birds’ habitat suitability maps to identify areas with high richness and priority for conservation. In another study, Ramírez-Albores et al.^27^ created SDMs for 180 bird species that are geographically restricted to Mesoamerica to identify high species richness areas and assess their representation within the network of protected areas. Thus, SDMs can be used to identify species-rich areas and facilitate conservation planning^18,32^.

Climate and land use changes threatened ecosystem services provided by biodiversity causing population decline^33–37^. Like other ecosystem services, pollination is also at risk of climate changes producing shift in avian distribution and local extinction of species able in pollination services^34,37^. For instance, Remolina-Figueroa et al.^38^ examined the co-distribution patterns of 12 Mexican endemic hummingbirds and 118 plants they used as nectar resources under climate change. They found that the relocation of species distributions can lead to mismatches between plants and their pollinators. Identifying species rich areas has important implications for conservation of biodiversity^39–42^. Availability of species distribution presence records at high-resolution and easily accessible environmental data allow us to map global distribution of different taxonomic groups^43–45^.

In this study, we aimed to develop a global habitat suitability map for sunbirds as a major group of avian pollinators. In addition, we quantified sunbird similarity within terrestrial biomes. We addressed the following questions:

1. Which geographical areas support the greatest diversity and suitability of sunbirds’ habitats?
2. What are the most important predictors of sunbirds global habitat suitability?
3. Which biomes have the highest diversity of sunbirds?

## Results

### Sunbirds’ habitat suitability and variable importance

**Fig. 1:**
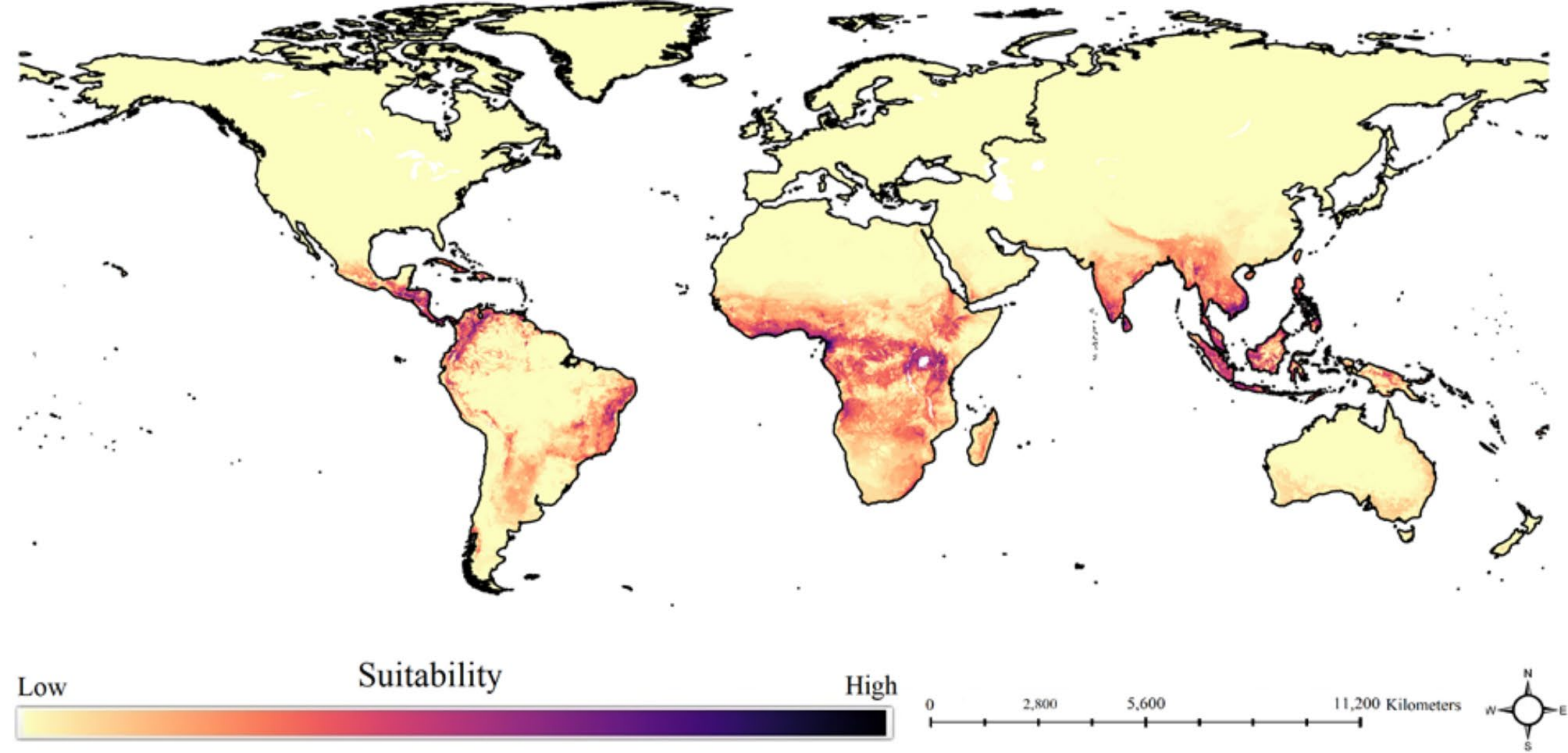
Global habitat suitability of sunbirds based on distribution of 124 species. Map was generated using QGIS 3.4.1 (http://www.qgis.org).

**Fig. 2:**
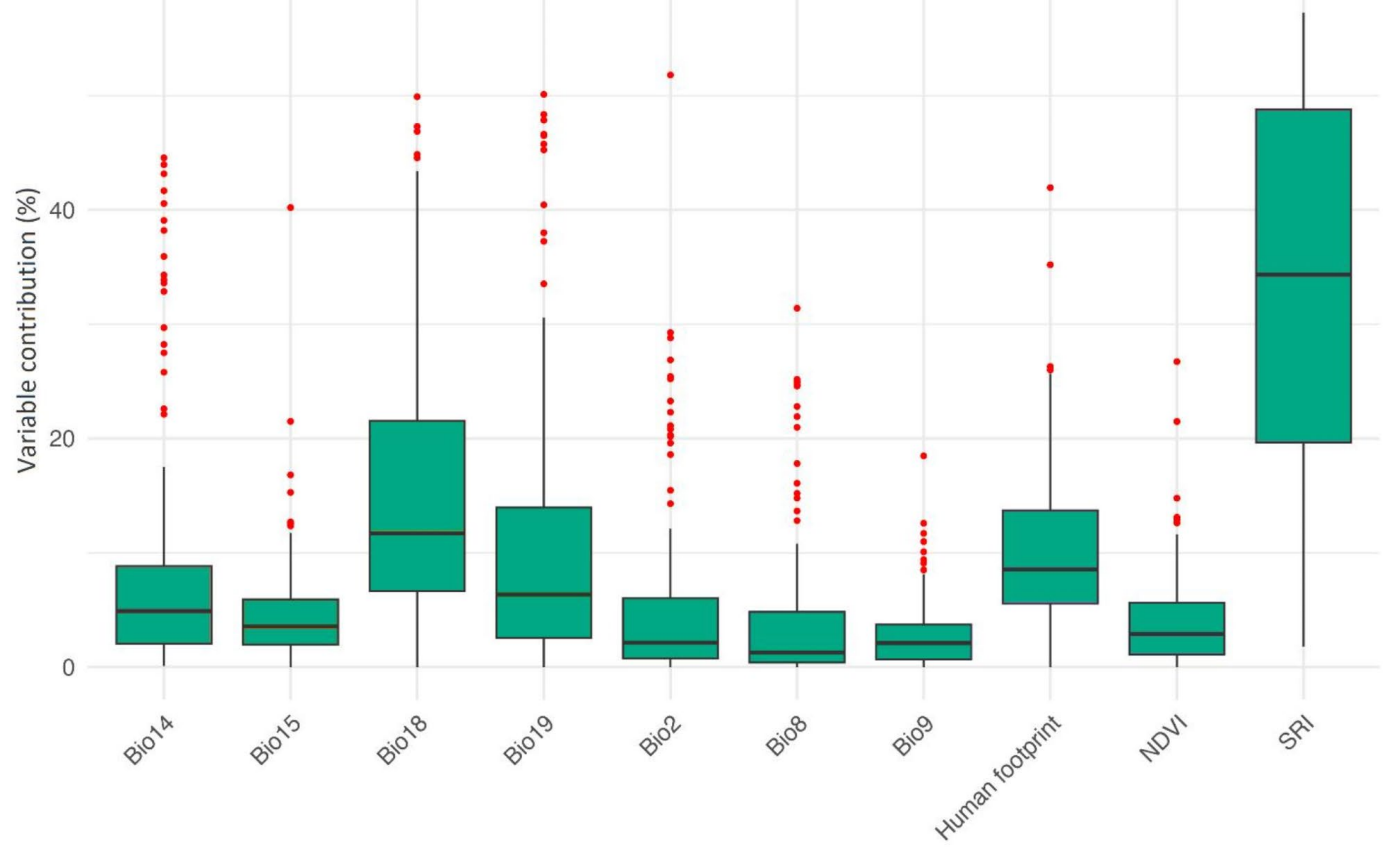
Boxplot of the variable contribution in predicting sunbirds habitat suitability. Variables contribution averaged across the 124 sunbird species. Variables’ abbreviations: Mean Diurnal Range (Bio2), Mean Temperature of Wettest Quarter (Bio8), Mean Temperature of Driest Quarter (Bio9), Precipitation of Driest Month (Bio14), Precipitation Seasonality (Bio15), Precipitation of Warmest Quarter (Bio18), Precipitation of Coldest Quarter (Bio19), Solar Radiation Index (SRI), and Human Footprint index (HF) and the Normalized Difference Vegetation Index (NDVI).

We found that all our models showed an AUC above 0.82. Sunbird SDMs highlighted that their habitat suitability is highest in Southeast Asia, western and central Africa. In contrast, North Africa and Southwest Asia show the lowest suitability for them (Fig. 1). Estimating the contribution of environmental variables in shaping global habitat suitability of sunbirds revealed that SRI, precipitation of warmest quarter and human footprint index were the most important drivers of sunbird species global habitat suitability (Fig. 2).

## Similarity within the terrestrial biomes

**Fig. 3:**
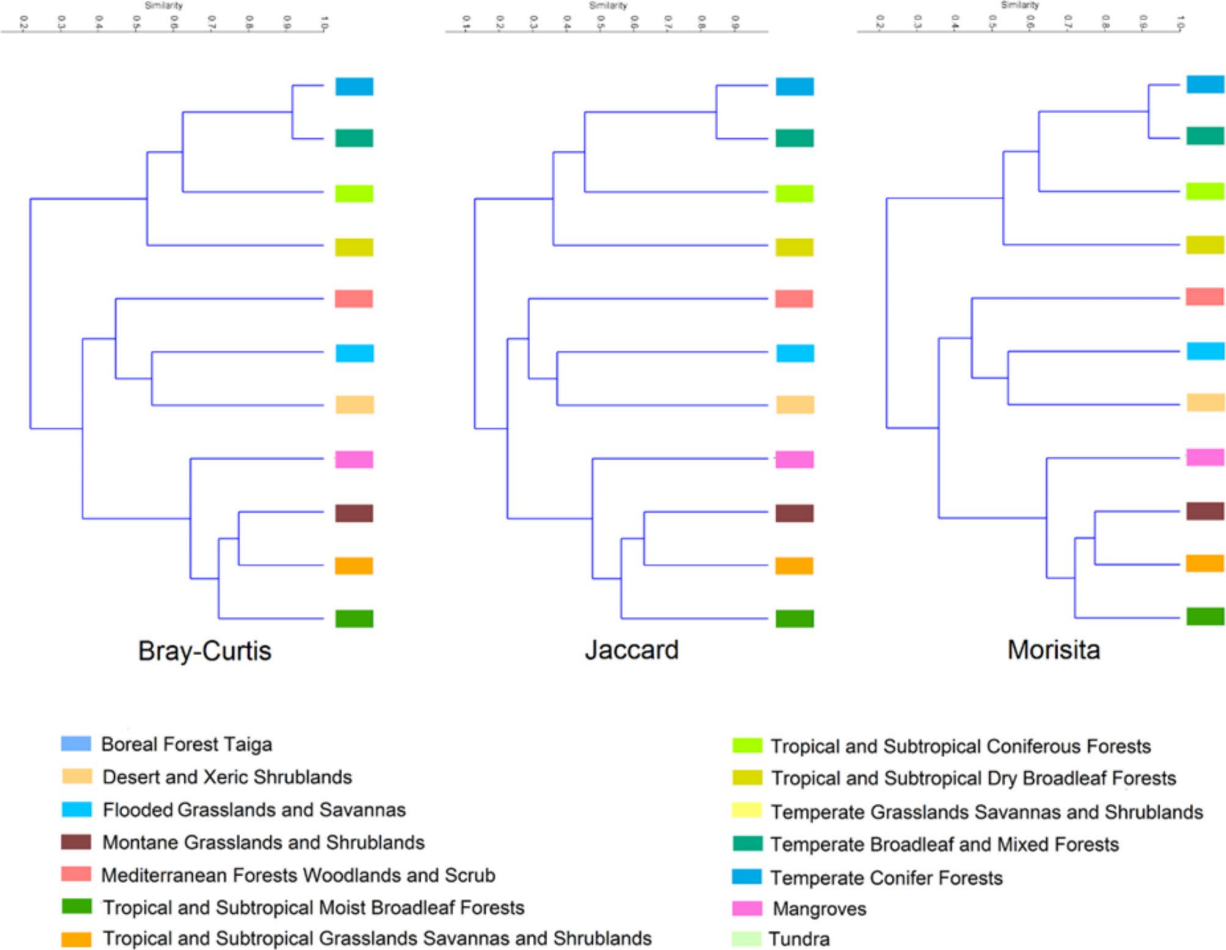
Similarity of terrestrial biomes based on sunbird assemblages calculated by using the Bray-Curtis, Jaccard and Morisita indexes. Figure was generated using QGIS 3.4.1 (https://www.qgis.org) and the Past software (Hammer et al., 2001).

We showed that the highest number of sunbirds live in Tropical and Subtropical Moist Broadleaf Forests and Tropical and Subtropical Grasslands Savannas and Shrublands biomes with 138 and 87 species, respectively (Table 1). Our analysis of sunbird similarity within the 11 terrestrial biomes highlighted that sunbird assemblage in the Mediterranean Forests Woodlands and Scrub biome are largely distinct from other biomes (Fig. 3). Temperate Broadleaf and Mixed Forests and Temperate Conifer Forests were the most similar biomes.

**Table 1:** Number of sunbird species recorded in terrestrial biomes.

Biomes	Number of species
Deserts and Xeric Shrublands	42
Flooded Grasslands and Savannas	28
Mangroves	66
Mediterranean Forests Woodlands and Scrub	14
Montane Grasslands and Shrublands	81
Temperate Broadleaf and Mixed Forests	12
Temperate Conifer Forests	12
Temperate Grasslands, Savannas and Shrublands	5
Tropical and Subtropical Coniferous Forests	20
Tropical and Subtropical Dry Broadleaf Forests	32
Tropical and Subtropical Grasslands Savannas and Shrublands	87
Tropical and Subtropical Moist Broadleaf Forests	138

## Discussion

We used SDMs to map the global potential distribution of sunbirds at high spatial resolution in order to identify areas with the highest suitability. The most suitable habitats for sunbirds are located in Tropical and Subtropical Moist Broadleaf Forests and Tropical and Subtropical Grasslands Savannas and Shrublands biomes.

Our results showed that SRI precipitation of warmest quarter and human footprint were the most influential predictors of sunbirds global habitat suitability. Solar radiation plays important role on avian distribution, ecology and behavior^46^. In fact, this variable affects the metabolic rate of animals and plants, producing different levels of physiological responses, thus influencing the egg hatching rates in birds, where high temperatures associated with low humidity ranges can lead to reduced reproductive success. Therefore, this predictor makes seasonality an important environmental filter for species distribution, resulting in changes in the composition and structure of communities^47^.

Precipitation of warmest quarter was the second most important variable in predicting sunbird global suitability. This variable is correlated to the nectar availability because generally more nectar is produced in areas with greater productivity and access/availability of water^48^. These results seem to confirm that sunbirds are highly dependent on plant species during warmest season, playing an important role in life cycle of several plant species. Human activities have devastating impacts on biodiversity through habitat destruction and land use changes^49,50^. Overall, we found that human footprint index is the third important predictor of global suitability of sunbirds. Major threats due to human impact to sunbirds include illegal hunting, urbanization, agriculture intensification, pollution, livestock, logging for timber, uncontrolled wildfires, collection of fuel wood, and conversion to agriculture accompanied by extensive burns. These threats are leading sunbird species toward extinction^51^. Understanding the subsequent effect of different disturbances on birds, and how the birds respond to each type and magnitude of human induced perturbations is fundamental to avifauna ecology, given that birds are good indicators of environmental quality^52^. Therefore, it is crucial to continuously monitor sunbirds diversity and abundance to see how these species adapt to ever-changing settings brought about by disturbances from human-caused habitat modification.

Global richness maps developed for different taxonomic groups of vertebrates are of utmost importance for conservation species diversity and abundance^53–55^. But for conservation of vertebrates’ ecosystem services, knowledge on species global distribution that provide ecosystem services is crucial^19,32,39,41,42^. In other words, we need specific maps showing the probability of the presence of each ecosystem service such as pollination, seed dispersal, and so on. Here we developed a specific global richness and habitat suitability map for avian pollinators, which provides the probability of presence of pollination services. We showed that Southeast Asia, western, and central parts of the African continent have the highest richness for sunbirds and consequently pollination services. Thus, these areas have high priority for conservation of avian biodiversity and, at the same time, pollination service. Further studies can plan to develop global maps for other ecosystem services like seed dispersal by modeling the global habitat suitability of species with seed dispersal service.

It is generally accepted that areas with higher species richness have a higher priority for conservation. But it also is important to identify and protect areas that contain the most unique assemblage of species that are not present in other regions^56,57^. For sunbirds, we showed that the Tropical and Subtropical Moist Broadleaf Forests biome have the highest species richness and thus have the highest priority for conservation of sunbirds. But we also propose the Mediterranean Forests, Woodlands, and Scrub biome as a high-priority biome for conservation because it has the most unique composition of sunbird species and is not similar to other biomes. Our global suitability model of sunbirds showed that despite suitable habitat, they never crossed the Atlantic Ocean^58^. We know that these suitable areas are occupied by another group of avian pollinators, hummingbirds^58^. Hummingbirds comprise the family Trochilidae that are found only in the Western Hemisphere^58,59^. Our richness map shows that avian pollinator richness should be highest in the Northwestern part of South America and Central America. Interestingly, the Northwestern of South America is identified to have the highest richness of hummingbirds^59^. These imply niche conservatism among the two groups of avian pollinators (e.g., sunbirds and hummingbirds) in tropical regions of the world^60,61^.

Climate change will cause shifts in avian distribution and can lead to local extinction of species with pollination services like sunbird and hummingbirds^34,37^. In addition, pollinators are particularly vulnerable to climate change because they depend on their host plant species^34,37^. Thus, it is necessary to study future distributions of pollinators such as sunbirds under climate and land use changes scenarios to be able to set proper conservation measures in areas that are predicted to experience higher changes in species distribution and composition.

Here we presented a global habitat suitability model for sunbirds and identified species-rich areas for their conservation. The model can serve as a baseline for large-scale conservation planning of pollination as an important ecosystem service. Since climate and land use change are happening at accelerated rates, the habitat suitability model developed for sunbirds can be used as a baseline to further document their impacts on sunbird richness. Pollination is an ecosystem service influencing the productivity of plants across the globe^62^. While we presented a global habitat suitability for a major group of avian pollinators, little is known about vertebrate pollinators global habitat suitability. Thus, we need global habitat suitability maps for other groups of pollinators to be able to conserve global diversity of pollinators and pollination. In this regard, SDMs are very practical tools in creating global-scale habitat suitability maps for vertebrate pollinators.

## Materials and methods

### Presence records

**Fig. 4:**
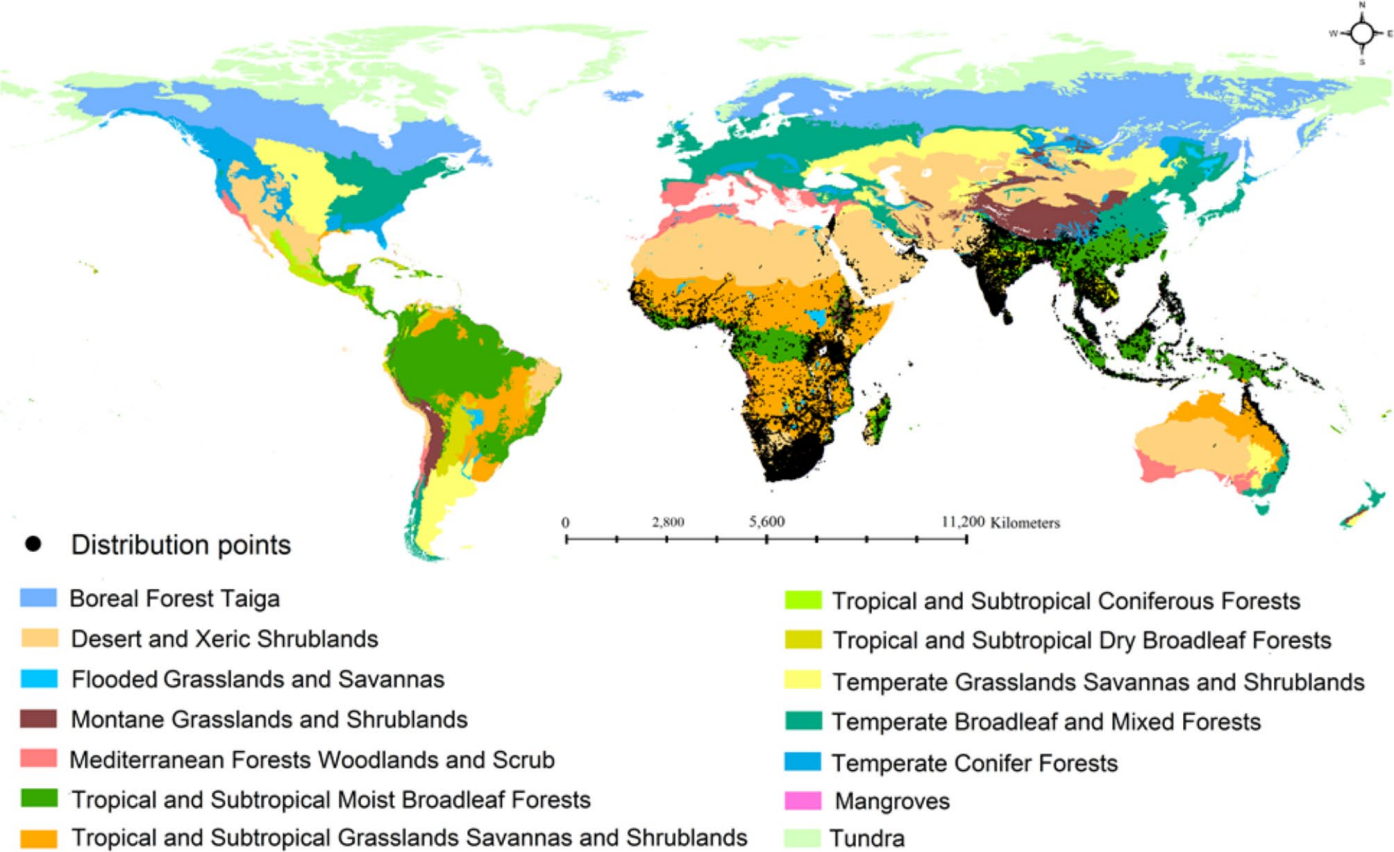
Global distribution map of sunbirds along with terrestrial biomes. The map was produced based on distribution records of 147 sunbird species. Map was generated using QGIS 3.4.1 (https://www.qgis.org).

We used the Handbook of the Birds of the World (HBW) and BirdLife Taxonomic Checklist for mapping sunbirds global suitability^16^. Our checklist includes 147 sunbird species for which we collected species distribution records obtained by the GBIF^63^, eBird, VertNet and iNaturalist databases using the spocc R package^64^. Since, we obtained distribution records from multiple sources, we carefully examined each species distribution data by mapping them in DIVAGIS 7.5^65^ to identify and remove outliers. We removed duplicates using ENMTools^66^. We obtained 1,947,560 distribution records for all sunbird species (Fig. 4). All species (147) distribution records were used to quantify the similarity of sunbirds among the terrestrial biomes. However, after removing outliers, duplicates and thinning distribution records to 5 km, we were able to model habitat suitability for 124 species.

### Environmental predictors

To model the sunbirds, we used the following environmental variables characterizing climate, topography, vegetation and anthropogenic conditions: Mean Diurnal Range (Bio2), Mean Temperature of Wettest Quarter (Bio8), Mean Temperature of Driest Quarter (Bio9), Precipitation of Driest Month (Bio14), Precipitation Seasonality (Bio15), Precipitation of Warmest Quarter (Bio18), Precipitation of Coldest Quarter (Bio19), Solar Radiation Index (SRI), the Normalized Difference Vegetation Index (NDVI), and human footprint index^67^. For the predictor variables, we chose biologically relevant variables and removed some correlated variables^68^. Climatic variables were downloaded from WorldClim 2.1^69^. SRI was obtained from the Shuttle Radar Topography Mission (SRTM) elevation model^70^. Human footprint index^71^ was used to quantify anthropogenic impact on the habitat suitability of the species (Venter et al. 2016a). This index was created by combining data on the extent of built environments, population density, electric infrastructure, crop lands, pasture lands, roads, railways, and navigable waterways^72^. To avoided collinearity among the variables a variance inflation factor (VIF)^73^ was calculated in the ‘usdm’ package^74^ in R software^75^. VIF values for the variables were < 10^18^ and no collinearity was found among them. All environmental variables were prepared at 5 km spatial resolution.

## Species distribution model

To build habitat suitability models for 124 sunbirds, we used Maxent because it is known to perform with high predictive accuracy, stability, and sensitivity^76^. Maxent not only performs better than other modeling approaches but also ensemble approach^76,77^. To properly parameterize Maxent, we used the Kuenm R package^78^. We employed this package to create candidate models with multiple combinations of regularization multipliers, feature classes, and sets of variables. Then, the best parameters for modeling were selected based on the statistical significance, predictive power, and model complexity^78^. This package uses Maxent to model target species^78,79^. The performance of the 124 habitat suitability models was assessed using the Area Under the Curve (AUC) metric of Receiving Operator Characteristic (ROC) curve^80^. AUC is a well-known model performance metric in SDM studies^18^. Values close to 0.5 suggest that the model has no predictive ability while values close to 1 show perfect predictive ability^80^. In this study, ROC plots were developed by using 80% of occurrence data for each species in model trainings and the remaining 20% as independent data in model testing.

Finally, we overlapped all the habitat suitability maps for each of 124 sunbirds species to obtain a global richness map for sunbirds in raster package in R environment^75^.

## Similarity of sunbirds assemblages among the terrestrial biomes

We mapped all 147 sunbirds distribution and overlayed it with world terrestrial biomes^81^ using QGIS 3.4.1 (https://www.qgis.org). Based on collected occurrence data, sunbirds are distributed in the following 12 biomes: Deserts and Xeric Shrublands, Flooded Grasslands and Savannas, Mangroves, Mediterranean Forests Woodlands and Scrub, Montane Grasslands and Shrublands, Temperate Broadleaf and Mixed Forests, Temperate Conifer Forests, Temperate Grasslands, Savannas and Shrublands, Tropical and Subtropical Coniferous Forests, Tropical and Subtropical Dry Broadleaf Forests, Tropical and Subtropical Grasslands Savannas and Shrublands, Tropical and Subtropical Moist Broadleaf Forests (Fig. 4). We created a matrix of all sunbird species presence and absence within the above-mentioned biomes using QGIS 3.4.1 (https://www.qgis.org). Then we used the Jaccard, Bray-Curtis, and Morisita similarity indices in the Past software^82^ to estimate similarity of sunbirds’ assemblages among the biomes.

## Data Availability

The datasets generated and analysed during the current study are available from the sources described in the manuscript. Occurrence data can be accessed at https://doi.org/10.15468/dl.9bcefj.
